# Development of a PLA Polymer-Based Liquid Filler for Next-Generation Aesthetics

**DOI:** 10.3390/ijms26052369

**Published:** 2025-03-06

**Authors:** Ji Hyun Sung, Na Jeong Park, Jeong Eun Park, Hye Sung Yoon, Ji Hyeon Baek, Helen Cho, Ji Hoon Park

**Affiliations:** Samyang Holdings Biopharmaceutical Group R&D Center, Seongnam 13488, Republic of Korea; jihyun.sung@samyang.com (J.H.S.); najeong.park@samyang.com (N.J.P.); jeongeun.park@samyang.com (J.E.P.); hyesung.yoon@samyang.com (H.S.Y.); jihyeon.baek@samyang.com (J.H.B.); helen.cho@samyang.com (H.C.)

**Keywords:** dermal filler, skin rejuvenation, biodegradable polymer, PLA filler, phagocytosis, collagen stimulator

## Abstract

In regard to both natural aging and photoaging caused by UV radiation, a decrease in skin collagen and elastin fibers results in the loss of soft tissue volume. Biodegradable polymer fillers have been used to overcome this problem, but the slow rate of reconstruction and particle agglomeration has limited this approach. The DMSB01 filler, which consists of poly d-l-lactic acid (PDLLA) with a methoxy polyethylene glycol (mPEG) initiator, was created to address this issue. In this study, we assessed the reconstruction and dispersion of the DMSB01 filler in vitro, as well as its effect on collagen expression in rats. DMSB01 showed rapid reconstruction and excellent dispersion stability; gelation occurred within 5 min at 37 °C and remained stable. In an animal model, DMSB01 induced M2 macrophages, Transforming growth factor beta (TGF-β) expression, and significantly increased collagens I and III. Collagen recovery and wrinkle improvement were confirmed by the aging and photoaging models, and hematoxylin and eosin (H&E) staining was used to demonstrate the safety and biodegradability of DMSB01. DMSB01 was effective in terms of inducing collagen production and improving skin aging, and shows promise as an innovative ingredient to overcome the limitations of existing fillers.

## 1. Introduction

For the last few years, the beauty market has focused on ‘anti-aging’, emphasizing the idea of aging slowly, beautifully, and healthily [1,2,3]. During aging, the skin becomes thinner and loses its elasticity, leading to drooping, loss of volume, and wrinkles [4,5]. Cosmetic fillers are a simple method for solving these issues without surgery. The most commonly used filler is hyaluronic acid (HA), a naturally occurring glycosaminoglycan that is widely distributed in the extracellular matrix [6].

However, HA fillers mainly help to restore facial volume; therefore, biodegradable polymer fillers have been developed for the purpose of regenerating skin collagen fibers that have been lost during aging. These fillers are made using existing biocompatible polymers, according to which insoluble polymers are processed into microscopic particles and dispersed in a viscous excipient or thickening agent [7]. For example, a typical formulation consists of Polycaprolactone (PCL) or Polylactic acid (PLA) that is 20–100 μm in diameter dispersed in HA, carboxymethylcellulose (CMC), or a glycerine solution [8]. However, these formulations can cause inconveniences during the procedure due to microscopic particles blocking the syringe needle and inconsistent tissue restoration effects due to uneven particle dispersion [9,10,11].

DMSB01 was developed to overcome these limitations. DMSB01 is a lyophilized formulation of mPEG-PDLLA and D-mannitol, which is made by polymerizing PDLLA, a polymeric filler material, with methoxy polyethylene glycol (mPEG) as an initiator.

Moreover, mPEG is widely used in biomedical applications. Over the past several decades, block copolymers composed of mPEG and biodegradable polyester segments have garnered significant interest in biomedical fields. Injectable hydrogels, formed from hydrophobic polymers and block copolymers, have been extensively studied, making them suitable materials for filler formulations [12,13,14]. PDLLA is a biodegradable, biocompatible polymer that induces TGF-β secretion from macrophages in the body, and its degradation product, lactic acid, is known to induce collagen formation in fibroblasts [15]. In addition to this, mPEG-PDLLA has been approved by the FDA as a drug delivery vehicle in the form of polymeric micelles [16,17]. Additionally, in vivo studies evaluating its biocompatibility, in microsphere and hydrogel forms, have demonstrated its safety [18,19]. D-mannitol is a freeze-drying additive, known to enhance the crystallinity of amorphous polymers, thereby increasing their resistance to collapse during the drying process [20,21,22].

DMSB01 has shown consistent dispersion stability in the absence of excipients, owing to the effects of hydrophilic mPEG. Therefore, we hypothesized that when DMSB01 is injected into the body as a liquid emulsion rather than as a solid, it will remain in the body in a gelated form, because of its temperature sensitivity. It was also important to test whether the remaining hydrophobic polymer, PDLLA, induced collagen formation.

The overall objective of this study was to assess the ease of DMSB01 injection and its efficacy in regard to collagen induction and wrinkle improvement. We performed assessments in parallel with Rejuran^®^ (Pharma Research, Gangneung, Republic of Korea), a liquid product that is known to be effective in stimulating collagen synthesis. Polynucleotide (PN), the main ingredient in Rejuran^®^, extracted from salmon gametes, has been reported to stimulate fibroblasts, which increase collagen synthesis and skin elasticity [23].

First, we evaluated the ability of the polymer filler to pass evenly through a thin needle and measured its extrusion force. Next, we assessed the in vivo biodegradation and collagen stimulation effects of DMSB01 and investigated the underlying mechanisms of these effects. Finally, we assessed the wrinkle improvement effects, using an animal model of UV-induced photoaging. Through this process, we developed a liquid filler from a PDLLA polymer that is capable of inducing collagen synthesis.

## 2. Results

### 2.1. Reconstruction and Dispersion Stability of DMSB01

**Figure 1:**
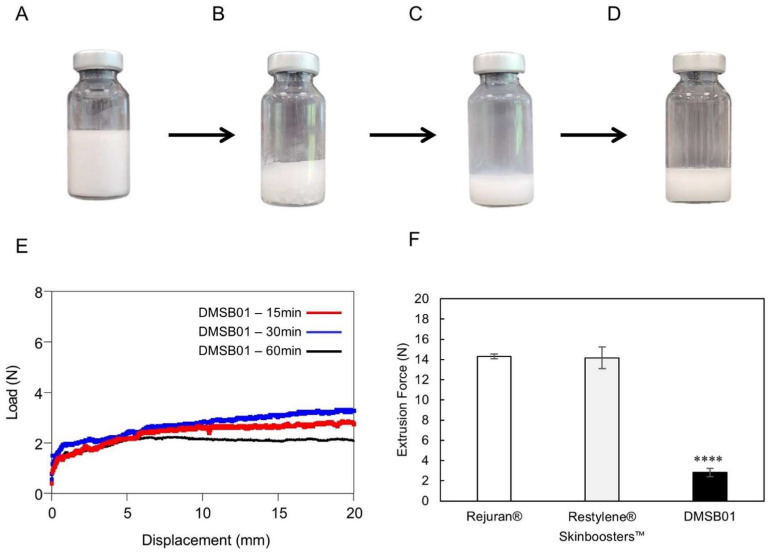
Optical images of DMSB01 reconstruction process and injection force. (**A**–**E**) DMSB01 vial photographs and graphs: (**A**) DMSB01 product; (**B**) after adding 3 mL of water for the injection; (**C**) photograph taken immediately after reconstruction; (**D**) reconstruction after 60 min; (**E**) graph of DMSB01 extrusion force 15, 30, and 60 min after reconstruction; (**F**) extrusion force graphs for Rejuran^®^, Restylene^®^ Skinboosters™, and DMSB01 (*n* = 3/group); **** *p* < 0.0001 (vs. first bar), Tukey’s multiple comparison test, and one-way ANOVA.

DMSB01 was reconstructed from a freeze-dried formulation (Figure 1A) by adding 3 mL of the injection solvent. The reconstruction speed is an important factor, as a faster reconstruction time enables earlier use, without the need to wait; however, the dispersion of the filler must also remain stable after reconstruction to enable injection without blockage of the syringe needle.

To test the reconstruction time and dispersion stability of DMSB01, we verified its appearance and measured its extrusion force. Figure 1B shows the appearance of DMSB01 immediately after adding water for the injection, which dissolves, as shown in Figure 1C, and when left for 1 h, as shown in Figure 1D; stable dispersion is maintained, without phase separation.

The dispersion stability was verified by measuring the extrusion force. When the solution is evenly and stably dispersed, viscosity differences are minimized in regard to all the syringes, and the extrusion force profile will be homogeneous. Figure 1E shows a graph of the extrusion force at 15 min, 30 min, and 1 h after reconstruction, demonstrating that the formulation was extruded with a consistent force, without blockage of the syringe needle throughout the entire time. This means that DMSB01 maintains dispersion stability even when time passes after reconstruction. Moreover, when we compared the peak extrusion force with that of the commercially available liquid skin boosters Rejuran^®^ and Restylene^®^ Skinboosters™ (Galderma, Lausanne, Switzerland) (Figure 1F), the peak extrusion force of DMSB01 (2.8 ± 0.4 N) was approximately five times lower than that of Rejuran^®^ (13.4 ± 1.5 N) and Restylene^®^ Skinboosters™ (14.2 ± 1.1 N).

### 2.2. Thermal Gelling Behavior of DMSB01

**Figure 2:**
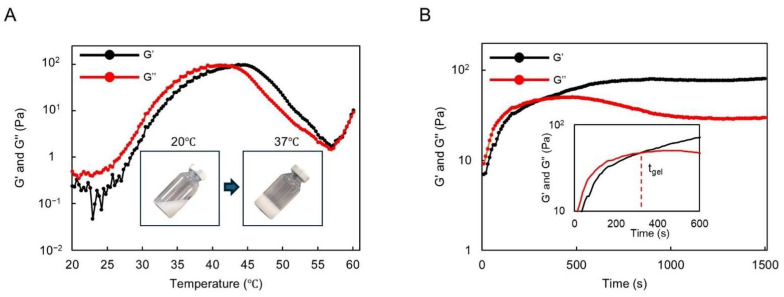
Thermogelling behavior assay and rheological analysis results: (**A**) graph of the storage modulus (G′) and loss modulus (G″) of DMSB01 for varying temperatures; (**B**) graph of the storage modulus (G′) and loss modulus (G″) at 37 °C over time.

We observed the sol–gel transition of DMSB01 through the use of dynamic rheological analysis (Figure 2). DMSB01 shows sol characteristics, with a loss modulus (G″) that is greater than the storage modulus (G′), up to 25 °C. As the temperature increased, both G′ and G″ increased, and the difference in the growth rate between G′ and G″ decreased at around body temperature. The sol–gel transition is defined as the point where G′ becomes larger than G″ [14,19], and the transition to a gel was observed at 43 °C (Figure 2A).

The gelation time of DMSB01 at 37 °C was also investigated (Figure 2B). Physically, a hydrogel was formed at 37 °C after approximately 300 s and, after gelation, G′ was maintained, whereas G″ decreased partially, resulting in improved phase persistence after gelation.

### 2.3. DMSB01 Increased Type 1 and 3 Collagen Expression via TGF-β Synthesis and the M2 Macrophage-Mediated Pathway

To assess the ability of DMSB01 to induce collagen production in the skin and investigate the cytokines and mechanisms involved, we administered the test substance intradermally into 2-month-old SD rats. TGF-β is a cytokine known to promote collagen formation by inducing the differentiation of fibroblasts into myofibroblasts [24]. M2 macrophages induce the expression of proteins required for the synthesis of collagen fibrils within the skin, and the number of M2 macrophages has been reported to be closely related to the amount of type I collagen specifically [25]. CD163 is reported to be a phenotypic marker of M2 macrophages in the skin [26].

**Figure 3:**
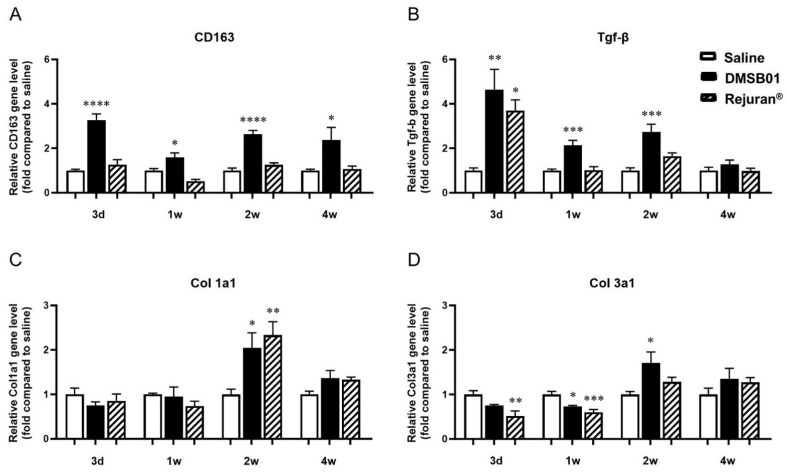
Changes in mRNA level of M2 macrophage-specific proteins CD163, TGF-β, and collagen following DMSB01 administration: (**A**) CD163; (**B**) TGF-β; (**C**) type I collagen; (**D**) type III collagen. The data are presented as the relative ratio to the mean value of the saline control at each time point and expressed as the mean ± SD (*n* = 6/group); * *p* < 0.05, ** *p* < 0.01, *** *p* < 0.001, **** *p* < 0.0001 (vs. first bar), Tukey’s multiple comparison test, one-way ANOVA.

We obtained tissue samples at 3 d, 1 w, 2 w, and 4 w after DMSB01 administration. RNA was extracted, and PCR analysis was performed for TGF-β, CD163, type I collagen (Col 1a1), and type III collagen (Col 3a1). CD163 expression was significantly higher than in the saline control at all the time points, and TGF-β expression was significantly higher at 3 d, 1 w, and 2 w (Figure 3A,B). Col 1a1 and Col 3a1 expression were significantly increased in the DMSB01-treated group compared to the saline control group 2 w after administration. Conversely, in the tissue treated with Rejuran^®^, an approved substance that was assessed in parallel, other than increased TGF-β after 3 d and increased Col 1a1 after 2 w, there were no significant changes in expression compared to the saline control group at any other time point (Figure 3C,D).

### 2.4. DMSB01 Enhanced the Expression of Type 1 Collagen in the Dermal Tissue of Aged Rats

After the PCR analysis confirmed that the dermal administration of DMSB01 significantly increased the collagen gene levels, we assessed its effects on collagen expression at the protein level. Many studies on SD rats have reported a significant decrease in skin collagen due to aging [27]. Particularly, while the collagen fractional synthesis rate in the heart, lungs, and skeletal muscle decreases to one-tenth of its former level due to aging, the collagen fractional synthesis rate decreases to one-twenty-fifth of its former level in the skin over the same period [28]. Type I collagen is a major structural protein in the skin, and many studies have used col1 as an aging-related marker [29].

**Figure 4:**
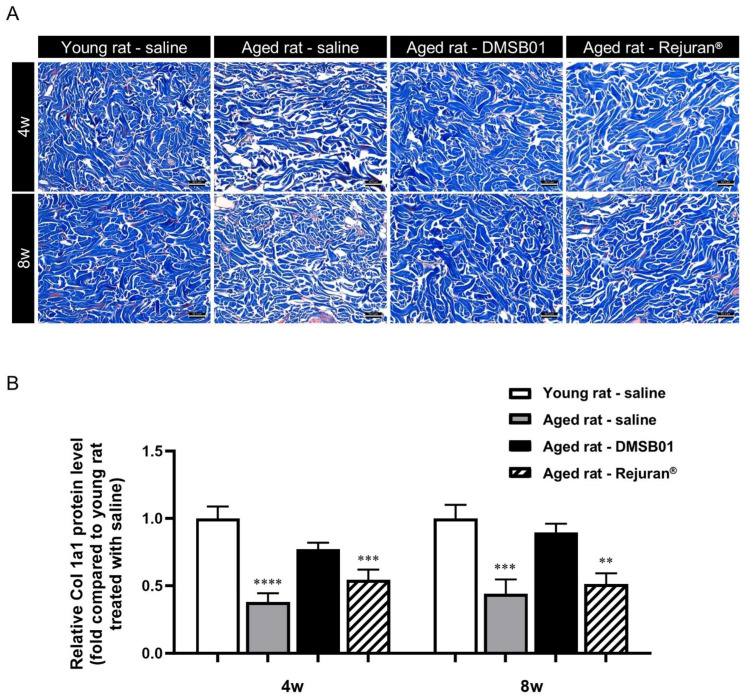
Changes in collagen after DMSB01 administration in aged rats: (**A**) Masson’s trichrome staining of injected dermal tissue (scale bar: 62.5 µm); (**B**) type I collagen ELISA analysis of injected dermal tissue. The data are presented as the ratio relative to the mean value of the young rat–saline group at each time point and are expressed as the mean ± SD (*n* = 6/group); ** *p* < 0.01, *** *p* < 0.001, **** *p* < 0.0001 (vs. first bar), Tukey’s multiple comparison test, one-way ANOVA.

To investigate the effects of DMSB01 administration on decreased skin collagen levels due to aging, we administered equal doses of 100 µL of DMSB01 intradermally into both young and aged rats and obtained tissue samples 4 and 8 w after treatment. To inspect the collagen fibers, we performed MT (Masson’s trichrome) staining. In the control rats treated with saline, we observed dense collagen formation in the dermis of young rats at both 4 and 8 w, while collagen in the dermis of aged rats showed a loose distribution. This demonstrated the decreased collagen in the dermis due to aging, as expected. When aged rats were treated with DMSB01, the collagen in the dermis was denser than that of the rats treated with saline, indicating that DMSB01 enhanced collagen synthesis. In rats treated with Rejuran^®^, the level of collagen was similar to that in aged rats treated with saline at both 4 and 8 weeks (Figure 4A).

After investigating the dermal collagen expression patterns due to aging and test substance administration using tissue slides, we performed a Col-1 ELISA kit analysis using tissue lysates from SD rats to quantify the collagen protein expression levels in the skin. The collagen protein expression levels were significantly lower in aged rats treated with saline or Rejuran^®^ at 4 and 8 w than saline-treated young rats. However, aged rats treated with DMSB01 showed increased collagen protein expression compared to saline-treated aged rats at both 4 and 8 w, and also showed no significant difference compared to saline-treated young rats (Figure 4B).

### 2.5. DMSB01 Improved the Wrinkles and Dermal Thickness in UVB-Photoaged Rats

We investigated the wrinkle improvement effects of DMSB01 using an in vivo model of chronic photoaging, induced by 8 weeks of UVB exposure. Prolonged exposure to sunlight causes photoaging of the skin, resulting in dryness, wrinkles, and pigmentation. UVB, with more energy than UVA and UVC, is mostly absorbed by epidermal cells [30], and is known to cause over 90% of cases of erythema, inflammation, and skin cancer related to UV exposure [31]. In rat skin, UVB radiation causes more rapid and pronounced damage than UVA, and continuous UVB irradiation induces photoaging, which causes changes such as exacerbation of wrinkles and thinning of the dermal layer [32].

Photoaging was induced with progressively increasing doses to mimic the gradual photodamage seen as a result of chronic UV exposure. We selected the dose based on the observed skin responses of the animal upon UV exposure and photoaging was induced through eight weeks of UVB irradiation [33,34]. To investigate the changes after the intradermal administration of DMSB01, we used PRIMOS, a three-dimensional skin measuring device, to evaluate wrinkle-related indices after 4 and 8 w. Additionally, we prepared tissue slides to measure dermal thickness. For comparison, we included a negative control group that did not undergo UVB-induced photoaging and a saline-treated control group that had been exposed to UVB radiation.

**Figure 5:**
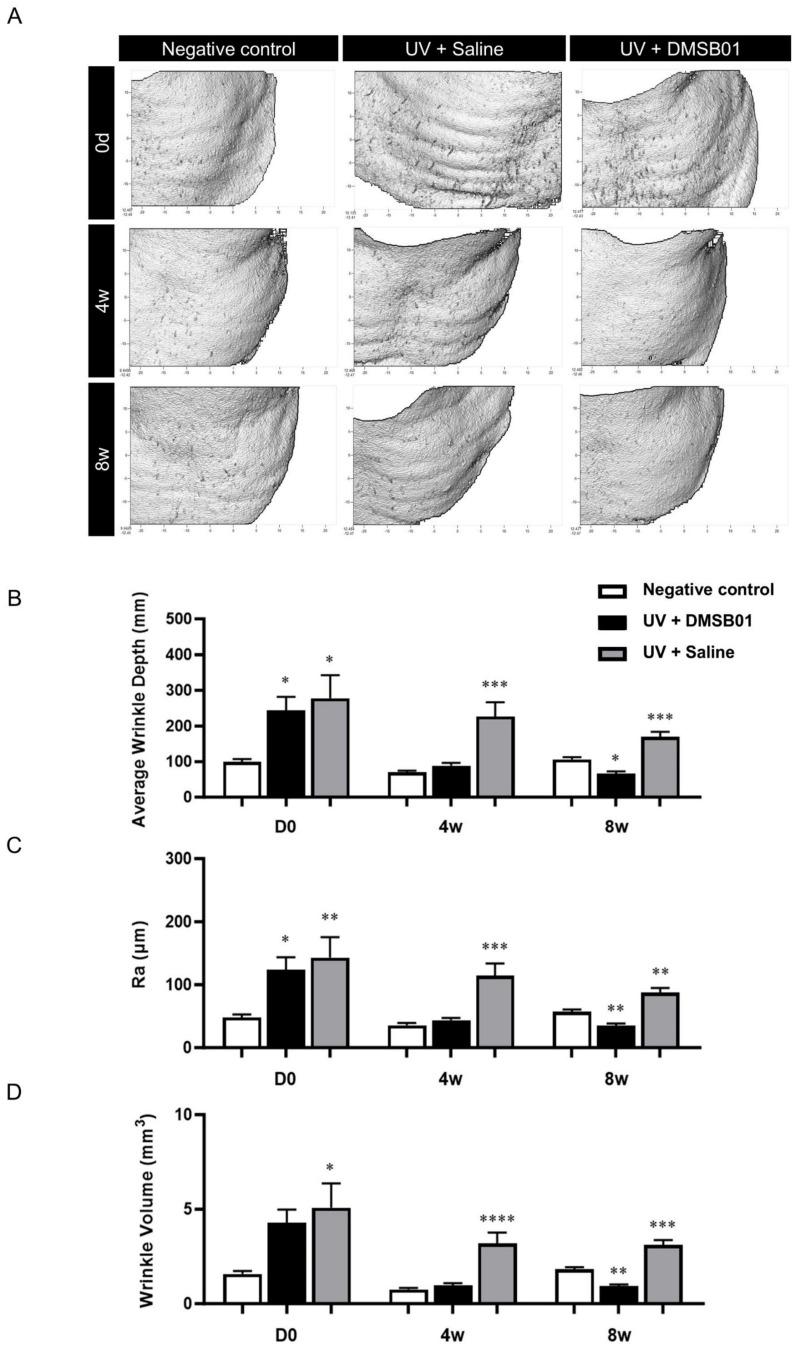
Changes in wrinkle indices following DMSB01 administration in the photoaging model: (**A**) representative PRIMOS images for each time point; (**B**–**D**) the results for the three wrinkle indices, namely (**B**) average wrinkle depth, (**C**) Ra and (**D**) wrinkle volume. Data are presented as the mean ± SD; * *p* < 0.05, ** *p* < 0.01, *** *p* < 0.001, **** *p* < 0.0001 (vs. first bar in each group), Tukey’s multiple comparison test, one-way ANOVA.

First, macroscopic analysis, using PRIMOS to capture images of the severity of wrinkles at the site of administration on the backs of hairless rats, showed that rats irradiated with UVB formed more wrinkles than the negative control. The rats in the negative control group showed no change in their wrinkles over 8 w; however, the rats treated with DMSB01 showed significant improvements in regard to their back wrinkles after 4 w, and this effect was maintained up to 8 w (Figure 5A). To better quantify these results, we analyzed three wrinkle measurement indices. After 0 d (before treatment), the average wrinkle depth (mm), roughness (Ra; µm), and wrinkle volume (mm^3^) were all higher in the UVB-photoaged rats compared to the negative controls, confirming that UV irradiation caused wrinkle formation. Among the UVB-photoaged rats, the saline-treated group showed no significant changes in the three wrinkle indices up to 8 w, whereas the rats treated with DMSB01 showed significant decreases in all three wrinkle indices after 4 w compared to those before treatment, indicating a significant improvement in their wrinkles. These changes remained consistent up to 8 w (Figure 5B–D).

**Figure 6:**
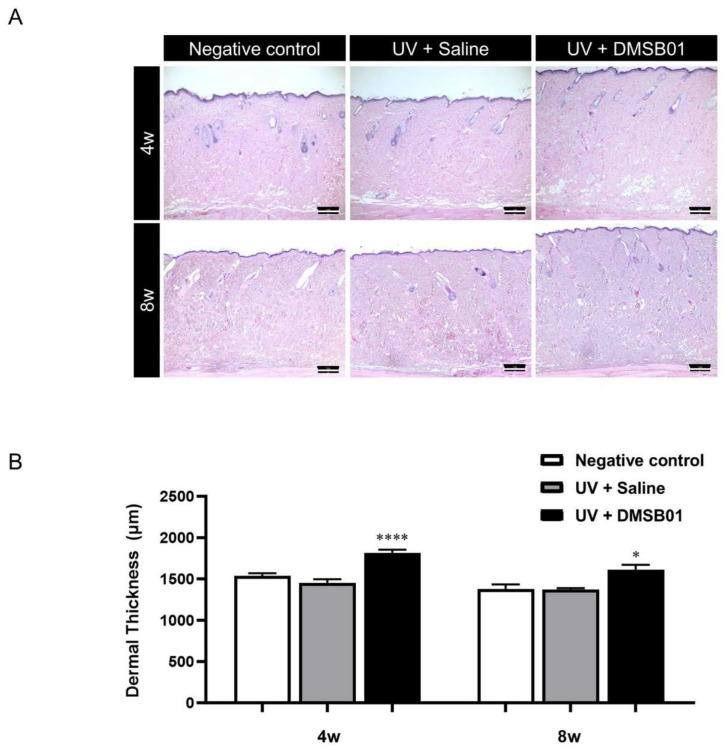
Changes in dermal thickness following DMSB01 administration in the photoaging model: (**A**) representative images after H&E staining for each time point (scale bar: 20 µm); (**B**) results of dermal thickness measurement using ImageJ software (version 1.53e). Data are presented as the mean ± SD; * *p* < 0.05, **** *p* < 0.0001 (vs. second bar), Tukey’s multiple comparison test, one-way ANOVA.

Next, in order to perform a microscopic analysis of the skin changes due to intradermal DMSB01 administration in photoaged rats, we harvested skin tissue from the rats in each test group at 4 and 8 w. All the skin tissue samples were fixed in formalin, embedded in paraffin blocks, sectioned with a thickness of 3 μm, prepared as tissue slides, and stained with hematoxylin and eosin (H&E), for examination using a light microscope. We observed thickening of the dermis at both 4 and 8 w after intradermal DMSB01 administration, compared to the saline-treated group (Figure 6A). Additionally, when we used ImageJ software (version 1.53e) to measure the dermal thickness of each tissue sample, the photoaged rats treated with intradermal DMSB01 showed significantly increased dermal thickness compared to the saline-treated rats after both 4 and 8 w (Figure 6B).

### 2.6. DMSB01 Was Safely Degraded After Dermal Injection

DMSB01 is a PDLLA-based dermal filler developed to induce collagen synthesis and improve wrinkles, following intradermal administration. In various in vivo rat models, we demonstrated that the intradermal administration of DMSB01 was effective in increasing collagen production and improving wrinkles in a UV-photoaging model. However, the ideal filler should not only demonstrate efficacy, but also biodegrade safely within the intended time, without causing any toxic side effects in regard to the surrounding tissues. To investigate the effects of degradation and the pattern of biodegradation after the intradermal administration of DMSB01, we collected skin tissue samples from the site of administration in SD rats at six time points from 1 d to 16 w. All the tissue samples were prepared as slides, stained with H&E, and subjected to a histological evaluation, using a light microscope.

**Figure 7:**
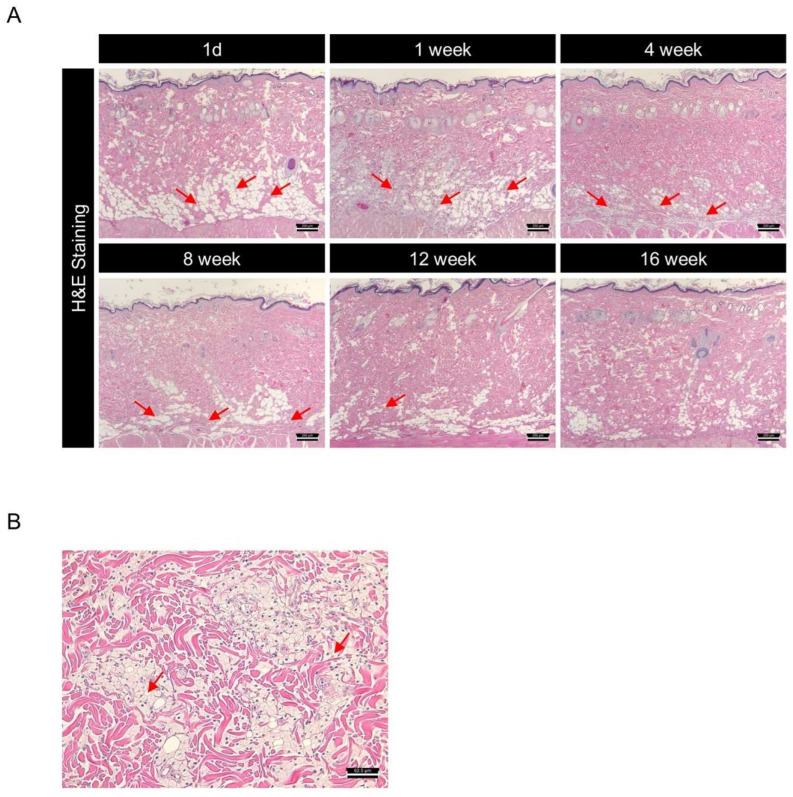
Investigation of biodegradation patterns and tissue responses to DMSB01 using hematoxylin and eosin (H&E) staining: (**A**) changes over time in the tissue where DMSB01 was administered, scale bar 250 µm; (**B**) DMSB01 was observed inside the histocytes after 1 week, scale bar 62.5 µm. Red arrow: DMSB01.

One day after administration, DMSB01 was mostly observed as clear vacuoles in the dermis, after which it was observed inside histiocytes, a type of phagocyte, up to 4 w. Thereafter, the test substance slowly degraded and was partially observed after 8 w, but it was mostly degraded by 12 w and completely degraded by 16 w (Figure 7A). When we examined the tissue changes at the site of administration, from 1 d after DMSB01 treatment, there were foamy histiocytes in the deep dermis, panniculus muscle layer, and subcutaneous layer, at a slight level. From 1 to 8 w, the distribution ranged from focal to diffuse. Inflammatory cells were observed at minimal levels in the deep dermis and subcutaneous layer, from 3 to 8 w after administration. At 12 w, both histiocytes and inflammatory cells were only observed at minimal levels in the subcutaneous layer, and by 16 w, DMSB01 was completely biodegraded, and no residual tissue was observed (Figure 7A,B).

## 3. Discussion

Human skin undergoes a gradual decrease in the amount of collagen and elastin fibers present due to UV-induced photoaging or natural aging [35]. Skin fillers are used to alleviate the loss of soft tissue volume caused by a decrease in such fibers due to aging. HA injections have various biological effects, such as supplying moisture to the tissue [36] and antioxidation [37]. Additionally, HA simulates type 1 collagen production and fibroblast proliferation via mechanical stress [38,39]. Fibroblasts in the skin at the site of a HA injection have a long, stretched appearance and express a high level of type 1 procollagen [40]; however, the induction of collagen formation via mechanical stimulation is relatively short lived [41].

Polylactic acid (PLA) polymers are used to induce collagen synthesis, mostly poly-lL-lactic acid (PLLA) or PDLLA microspheres [15], which are enantiomers (chiral molecules). PLA can induce collagen production even after degradation, which differentiates it from other fillers that only aim to increase the facial volume [42]. After injection, PLA fillers induce the differentiation of macrophages into the M2 subtype, which is known to induce collagen production by increasing TGF-β expression in fibroblasts and is a biocompatible material that is hydrolyzed into water and carbon dioxide [43]. However, because of these hydrolytic properties, PLA fillers are manufactured in a freeze-dried formulation with no water, and to disperse the PLA microspheres in the solvent during reconstruction, carboxymethylcellulose (CMC) or HA is used as a thickening agent. When using these thickening agents during reconstruction, it takes an exceptionally long time to dissolve the thickener and disperse the microspheres. Moreover, the microspheres are hydrophobic and do not dissolve in water; therefore, there is a risk of adverse effects, due to particle agglomeration when the hydrophilic thickening agent is released into the body [9,10,11].

The purpose of this study was to analyze the physical properties and in vivo efficacy of DMSB01, a block copolymer containing PDLLA with methoxy polyethylene glycol (mPEG) as the initiator, to overcome the limitations of previous approaches. DMSB01 was manufactured in a freeze-dried state and showed a short reconstruction time and excellent dispersion stability, even in the absence of a thickening agent. In regard to the rheometer analysis, reconstruction to a liquid state was easy at room temperature, and gelation started within 5 min at body temperature (37 °C), enabling it to remain stable in the body, even after injection. This suggests that the hydrophilicity of mPEG allows it to disperse easily and maintain excellent stability when reconstituted in an aqueous solution. Furthermore, with the introduction of mPEG, the formulation exhibits temperature sensitivity, remaining in a liquid state at room temperature and forming a hydrogel near body temperature. This allows for formulation at room temperature and gelation and retention within the body upon injection. This enhances its usability compared to conventional PLA-based fillers [44,45].

PDLLA fillers have been reported to induce M2 polarization, leading to increased TGF-β expression by fibroblasts and, ultimately, stimulating collagen production [46]. We hypothesized that DMSB01, the main ingredient of which is mPEG-PDLLA, could induce collagen production via a route similar to that of PDLLA. For the control group, we used a PN filler, also in non-particle form, which is known to induce fibroblast migration and type 1 collagen production in human skin [47,48]. We used genetic analysis involving SD rats to confirm that within one week after DMSB01 administration, there was significantly increased expression of the M2 macrophage marker, CD163, and increased secretion of TGF-β, and we also confirmed that collagens I and III were expressed after 2 weeks. This suggests that DMSB01 has the physiological effect of inducing collagen production beyond simple volume-increasing fillers. In an aged SD rat model, DMSB01 restored the collagen levels similar to those observed in young SD rats. The ELISA analysis and MT staining showed that DMSB01 effectively alleviated the aging-related reduction in collagen. Additionally, in a UVB-induced photoaging model, DMSB01 improved the wrinkle depth and skin roughness, demonstrating its potential ability to promote the recovery of skin injuries due to photoaging. H&E staining was used to confirm the safety and biodegradability of DMSB01. The main component, mPEG-PDLLA, is known to degrade into lactic acid and H₂O, as its final decomposition products [49,50]. Lactic acid has a pKa of 3.8 [51,52], resulting in a low pH, and its accumulation as a degradation byproduct may induce inflammation. Therefore, inflammatory cells associated with biodegradation were examined. Up to three weeks after DMSB01 injection, phagocytosis by histiocytes, a type of phagocyte, was primarily observed, while inflammatory cells appeared after three weeks. This inflammation was likely induced by lactic acid generated during degradation and was observed at a minimal grade, persisting until 12 weeks post-injection. By 16 weeks, the formulation had fully degraded, with no observable effects on the surrounding tissues. These results suggest that DMSB01 is a biocompatible and a safe biodegradable filler. Although this study assessed tissue changes up to 16 weeks, further research is required to evaluate the potential long-term consequences of chronic inflammation that may arise from the accumulation of degradation byproducts.

Our study showed that DMSB01 is effective in regard to collagen synthesis and improving the skin’s condition, and that it has potential as an innovative material that can overcome the limitations of existing fillers. Notably, DMSB01 showed excellent efficacy and safety in aging and photoaging models, indicating that DMSB01 is likely to play a very important role in the fields of dermatology and regenerative medicine.

## 4. Materials and Methods

### 4.1. DMSB01 Preparation

Specifically, mPEG-PDLLA was synthesized by Samyang Holdings via ring-opening polymerization of mPEG (SinoPEG, XiaMen, Fujian, China) and D,L-lactide (Samyang Holdings, Gongju, Republic of Korea), using Sn(Oct)2 (Merck, Boston, MA, USA) as a catalyst. The DMSB01 was prepared through the dissolution of 400 mg of mPEG-PDLLA and 40 mg of D-mannitol (Roquette, France) in an aqueous solution, followed by freezing at −40 °C and lyophilization.

### 4.2. Characterization

#### 4.2.1. Reconstruction and Dispersion Stability

The reconstitution time and dispersion stability of DMSB01 was verified by measuring the extrusion force as a function of the standing time after reconfiguration to ensure that the needle extruded evenly and without clogging. Briefly, 3 mL of water for the injection (Daihan, Seoul, Republic of Korea) was added to DMSB01. The prepared dispersion was left for 15, 30, and 60 min, and then 1 mL was taken, and the extrusion force was measured by connecting a 33G syringe needle (Japan Bio Products, Tokyo, Japan). The extrusion force was measured using a Universal Test Machine (Intron 4465, Software-Series IX version 8.28), equipped with a load cell with a capacity of 50 kN. After loading the sample into the measuring module, the compressive strength was measured by pressing the syringe pusher at a constant 12 mm/min descending speed until all the contents were ejected, and the maximum value of the measured value was calculated as the extrusion force.

#### 4.2.2. Rheological Measurements

Rheological tests were performed with a Rheometer (MCR102e, Anton Paar, Graz, Austria), fitted with a 25 mm parallel plate geometry, with a gap distance of 0.8 mm. The temperature was controlled with a Peltier plate system and temperature sweep tests were performed with an oscillation temperature ramp of 20–60 °C, at a ramp rate of 1 °C/min. The storage modulus (G′) and loss modulus (G″) were measured as functions of the temperature. The data were collected under a constant strain of 1% and an angular frequency of 1.0 Hz. The gelation time of DMSB01 at 37 °C was also investigated, recording G′ and G″ as a function of time, and defined as the time at which G′ became higher than G″.

### 4.3. In Vivo Model

#### 4.3.1. Healthy Young Rat and Aged Rat Models

All the animal experiments were approved by Samyang Holdings’ Institutional Animal Care and Use Committee (IACUC) and conducted in an environment that minimizes animal suffering and complies with animal welfare laws and regulations, as required by the applicable guidelines (Approval No. SYAU-2321, SYAU-2401, SYAU-2421). Six-week-old male SD rats were obtained from Orient Bio, Inc. (Seongnam, Republic of Korea). The animals were housed in a controlled laboratory environment: a temperature of 24 ± 2 °C, a relative humidity of 50 ± 10%, and a 12-h light–dark cycle. They had unrestricted access to food and water. A minimum acclimation period of one week was provided prior to the start of the experiments. After the acclimation period, the rats were divided into two groups: 2-month-old young rats (*n* = 6/treatment group/time point) and 10-month-old aged rats (*n* = 3/treatment group/time point). Moreover, 100 µL of saline was used as a negative control; DMSB01, or Rejuran^®^ (Pharmaresearch, Gangneung, Republic of Korea) as a predicate control, was injected into the dorsal skin on the back of the rats, and skin tissues were then collected, according to the experimental design for each test.

For the qPCR analysis, skin tissues from the young rats were collected 3 days, 1 week, 2 weeks, and 4 weeks later (*n* = 6). For the ELISA and histological analysis, skin tissues from both the young and aged rats were collected at 4 weeks and 8 weeks (*n* = 6). All the animals were euthanized using CO₂ gas, and the injection sites were shaved and sterilized with 70% ethanol prior to the collection of skin tissues.

#### 4.3.2. UVB-Photoaging Rat Model

All of the animal handling procedures were conducted in accordance with the Guidelines for the Care and Use of Laboratory Animals and received approval from the Animal Ethics Committee at Ajou Advanced Medical Bio Research Institute IACUC (Approval No. 2023-0113). Seven-week-old HWY/Slc hairless male rats were obtained from Central Lab. Animal Inc., (Seoul, Republic of Korea) and a minimum one-week period of adaptation followed before treatment. Photoaging was induced through UVB irradiation, using a UV radiometer (VILBER, Marne-la-Vallée, France), and the frequency of UVB exposure was determined as 3 times per week for 8 weeks. The detailed dose of UVB exposure was 50 mJ/cm^2^ in the first 4 weeks, 75 mJ/cm^2^ for the following 2 weeks, and 100 mJ/cm^2^ for the last 2 weeks, with a total dose of 1650 mJ/cm^2^.

The photoaged animals were divided into two groups and intradermally injected into their back with saline or DMSB01, respectively, 100 µL each at 4 sites in a 2 cm × 2 cm area. Wrinkle formation was evaluated at 0 days (pre-injected), 4 weeks, and 8 weeks, and skin tissues were collected for analysis of the dermal thickness for all the rats at 4 weeks and 8 weeks. The present experiments were performed at the KBI (Korea Biomedical Research Institute, Seongnam, Republic of Korea).

### 4.4. RNA Quantitation

#### 4.4.1. RNA Extraction

The total RNA was extracted from the rat skin tissues, following the manufacturer’s instructions. In brief, the excised skin tissues were immersed in RNAlater™ solution (Invitrogen, Waltham, MA, USA), rinsed with cold PBS (Gibco, Waltham, MA, USA), and then homogenized in TRIzol™ reagent (Thermo Fisher Scientific, Waltham, MA, USA). RNA extraction was performed using an AccuPrep^®^ Universal RNA Extraction Kit (Bioneer, Daejeon, Republic of Korea). The quality, concentration, and purity of the extracted RNA were assessed using a NanoDrop One spectrophotometer (Thermo Fisher Scientific, Waltham, MA, USA), and the RNA Quality Number (RQN) and 28 S/18 S peaks were assessed using a 5200 Fragment Analyzer (Agilent, Santa Clara, CA, USA).

#### 4.4.2. cDNA Synthesis

Following the manufacturer’s instructions, the RNA (1 µg) was converted into cDNA using an AccuPower^®^ Rocketscript™ Cycle RT Premix (Bioneer, Daejeon, Republic of Korea). Briefly, the template RNA was mixed with the premix containing Oligo dT and RNase-free distilled water to a final volume of 20 µL. The mixture was then incubated in an AllInOneCycler™ PCR system (Bioneer, Daejeon, Republic of Korea) under the following conditions: 37 °C for 30 s, 48 °C for 4 min, and 55 °C for 30 s, repeated for 12 cycles, followed by a final incubation at 95 °C for 10 min to synthesize the cDNA.

#### 4.4.3. Quantitative Real-Time Polymerase Chain Reaction (qRT-PCR)

The expression of the target genes was quantified by qRT-PCR using an AccuPower^®^ Plus DualStar™ qPCR Master Mix (2×, 2.5 mL) (Bioneer, Daejeon, Republic of Korea), gene-specific TaqMan probes, and cDNA. In brief, each well in a 384-well plate contained 3 µL of 1:3 diluted cDNA, 7.5 µL of 2× master mix, 0.75 µL of 20× primers, and 3.75 µL of distilled water. Amplification was conducted using an Exicycler™ 384 Real-Time Quantitative Thermal Block (Bioneer, Daejeon, Republic of Korea) under the following conditions: an initial denaturation at 95 °C for 10 min, followed by 40 cycles at 95 °C for 15 s and 60 °C for 1 min. The relative gene expression was calculated using the comparative CT (∆∆CT) method, and the mRNA levels were normalized to Actb. The data were presented as the fold change relative to the first bar in each graph.

### 4.5. Protein Quantitation

#### 4.5.1. Protein Extraction

All the tissues were finely chopped using surgical scissors and then homogenized using a TissueRuptor II (Qiagen, Hilden, NRW, Germany) probe in 3.5 mL of DPBS (Gibco, Waltham, MA, USA), following 3 cycles of 15 s in ice and 10 s off ice. After homogenization, cell lysis was achieved through sonication via a 550 sonic Dismembrator (Thermo Fisher Scientific, Waltham, MA, USA) under the same conditions: 15 s on, 10 s off, for 3 cycles, in ice. The tissue homogenates were then centrifuged at 12,400 RPM for 15 min at 4 °C to obtain the supernatants, which were stored at −80 °C in a deep freezer until the ELISA analysis

#### 4.5.2. Enzyme-Linked Immunosorbent Assay (ELISA)

The expression of collagen was quantified through an ELISA using the Rat Collagen I ELISA Kit (LSBio, Seattle, WA, USA). In brief, a dilution ratio of 1:10 in regard to 100 µL of tissue lysates and 100 µL of the standard were applied to each well of the ELISA plate, in duplicate. After incubation at 37 °C, the plate was washed with wash buffer, then the detection antibody, HRP–Streptavidin conjugate and TMB Substrate were sequentially applied while incubating at 37 °C. The reaction was stopped by adding a Stop Solution, and the absorbance was measured at 450 nm, using a BioTek Synergy HTX Multimode Reader (BioTek, Seoul, Republic of Korea). The data were converted into protein concentrations using the Gen5 software(version 3.11). The protein levels were presented as the fold change relative to the value of the first bar in each graph.

### 4.6. Wrinkle Analysis

Wrinkle analysis was performed via the PRIMOS High Resolution^®^ system (Canfield, Parsippany, NJ, USA) at 0 days (pre-injected), 4 weeks, and 8 weeks for the UVB non-treated rats (negative control group) and UVB-treated rats. Under respiratory anesthesia, using 0.3% isoflurane, three parameters were measured: average wrinkle depth (mm), Ra (µm), wrinkle volume (mm^3^).

For the dermal thickness analysis, the rats were euthanized in a CO_2_ chamber and the injected skin tissues were excised at 4 weeks and 8 weeks after injection. The skin tissues were fixed in 10% neutral buffered formalin and embedded in paraffin. The paraffin-embedded skin tissue blocks were sectioned into 3 µm thick slides, then, hematoxylin and eosin (H&E) staining was performed. The slides were observed under an optical microscope (Carl Zeiss NTS Ltd., Oberkochen, Germany) and captured by the ZeMicroscope software iss Zen 3.4 Blue Edition (Carl Zeiss NTS Ltd., Oberkochen, Germany). The thickness of the dermal layer was quantified for all the stained slides using the ImageJ software (The National Institutes of Health, Bethesda, MD, USA. version 1.53e) program. The dermis thickness was measured at two points per section, 5 sections for each test article. The results were presented as the average dermal thickness for each test article at each timepoint.

### 4.7. Degradation Test

Seven-week-old SD rats were obtained from Orient Bio, Inc. (Seongnam, Republic of Korea) and were housed in a controlled laboratory environment. Following a sufficient acclimation period, 100 μL of DMSB01 was injected into the dorsal skin of the rats at 11 weeks of age. To evaluate the tissue response to DMSB01 administration, skin samples were collected at 1 day, 1 week, 4 weeks, 8 weeks, 12 weeks, and 16 weeks later and the skin tissues were sectioned with a thickness of 3.5 µm. All the slides were stained with hematoxylin and eosin (H&E), then the degradation and tissue response to DMSB01 following intradermal injection were evaluated.

### 4.8. Paraffin-Embedded Block Preparation and Sectioning

The skin tissues were fixed onto OHP film using a stapler, and then fixed in 4% paraformaldehyde (PFA; Sigma-Aldrich, St. Louis, MO, USA) for one week. After fixation, the tissues were gradually dehydrated using a tissue processor (Thermo Fisher Scientific, Waltham, MA, USA) by increasing the ethanol concentrations to 70%, 80%, 90%, and 100%. Following the removal of residual ethanol with Xylene, the tissues were embedded in paraffin. The paraffin-embedded blocks were sectioned to a thickness of 3.5 µm thickness using a microtome (Thermo Fisher Scientific, Waltham, MA, USA) and dried at 37 °C to prepare the tissue slides.

### 4.9. Histological Analysis

#### 4.9.1. Hematoxylin and Eosin (H&E) Staining

For the hematoxylin and eosin (H&E) staining, the slides were deparaffinized three times in Xylene for 7 min each. The slides were then immersed in ethanol at a concentration of 100%, 90%, 80%, and 70% for 3 min each to replace the Xylene and rehydrate the slides. After thoroughly rinsing with water, the slides were stained with hematoxylin for 2 min to stain the nuclei, followed by rinsing with running water to remove any excess staining solution. To remove the residual hematoxylin, the slides were immersed in 1% acetic acid for 2 min, followed by immersion in 0.5% ammonia for 2 min. The slides were then stained with eosin for 6 min to stain the cytoplasm. The slides were then dehydrated by immersing them in ethanol at a concentration of 70%, 80%, 90%, and 100%. Clearing was performed by immersing the slides in Xylene, three times for 5 min each. Finally, the slides were mounted for the histological analysis.

#### 4.9.2. Masson’s Trichrome Staining

For Masson’s trichrome staining (MT), the slides were deparaffinized three times in Xylene for 7 min each. The slides were then immersed in ethanol at a concentration of 100%, 90%, 80%, and 70% for 3 min each to replace the Xylene and rehydrate the slides. After thoroughly rinsing with water, the slides were fixed in Bouin’s solution at 56 °C for 1 h. Following rinsing with running water, the slides were immersed in a 1:1 mixture of hematoxylin A and B for 10 min to stain the nuclei, followed by rinsing with running water to remove any excess staining solution. The slides were stained with Scarlet Red-acid fuchsin solution for 10 min, followed by rinsing with running water. To decolorize the slides, they were immersed in a phosphomolybdic–phosphotungstic acid solution for 10 min. The slides were then stained with Aniline Blue for 5 min to stain the collagen fibers, followed by thorough rinsing with running water and immersion in 1% acetic acid for 2 min. The slides were dehydrated by immersing them in ethanol, with a concentration of 90% and 100%. Clearing was performed by immersing the slides in Xylene, three times for 5 min each. Finally, the slides were mounted for the histological analysis.

### 4.10. Statistical Analysis

For the RNA and protein quantification results, values exceeding the 95% range of the normal distribution were excluded through outlier testing using Minitab Statistical software (Minitab version 21.1.1.0, Inc., State College, PA, USA). The data were then presented as mean ± standard deviation (SD). Statistical comparisons were conducted using a one-way ANOVA in GraphPad Prism 10 (GraphPad Software Inc., San Diego, CA, USA). A *p*-value of less than 0.05 was considered statistically significant.

## 5. Conclusions

In this study, we introduced DMSB01, a new skin filler consisting of mPEG-PDLLA dispersed evenly in water, to resolve the specific problems associated with existing fillers. Our findings demonstrated that unlike existing products on the market, DMSB01 is easily reconstructed into a liquid at 25 °C and below, is injectable, and undergoes gelation in the body, effectively stimulating new collagen formation via the same mechanisms as PDLLA, thereby aiding skin regeneration. This study is significant because we have investigated the characteristics of hydrophilicity when introduced to a polymer used in fillers and have proposed new uses. In the future, clinical studies need to be conducted to verify the practical applications of this material and its long-term safety.

## Data Availability

Date is contained within the article.
